# Production and certification of BOTS-1: bovine muscle–certified reference material for incurred veterinary drug residues

**DOI:** 10.1007/s00216-023-04794-5

**Published:** 2023-06-16

**Authors:** Garnet McRae, Donald M. Leek, Juris Meija, Bryn Shurmer, Steven J. Lehotay, Joachim Polzer, Jeremy E. Melanson, Zoltan Mester

**Affiliations:** 1 National Research Council Canada, Metrology, 1200 Montreal Road, ON K1A 0R6 Ottawa, Canada; 2 Canadian Food Inspection Agency, 116 Veterinary Road, Saskatoon, SK S7N 2R3 Canada; 3 US Department of Agriculture, Agricultural Research Service, Eastern Regional Research Center, 600 East Mermaid Lane, Wyndmoor, PA 19038 USA; 4 German Federal Office of Consumer Protection and Food Safety (BVL), Mauerstrasse 39-42, 10117 Berlin, Germany

**Keywords:** Isotope dilution, LC–MS/MS, Mass spectrometry, Certified reference material, Veterinary drugs

## Abstract

A freeze-dried bovine muscle–certified reference material (CRM), known as BOTS-1 (DOI: 10.4224/crm.2018.bots-1), containing incurred residues of commonly used veterinary drugs was produced and certified for the mass fraction of eight veterinary drug residues. Value assignment was carried out using liquid chromatography tandem mass spectrometry (LC–MS/MS) methods in conjunction with isotope dilution and standard addition approaches involving stable isotope internal standards. Data from the National Research Council of Canada (NRC), Canadian Food Inspection Agency (CFIA), United States Department of Agriculture (USDA), and the Federal Office of Consumer Protection and Food Safety in Germany (BVL) were used for value assignment. Results for two drug residues were also obtained through an international inter-laboratory comparison CCQM-K141/P178 organized under the auspices of the International Bureau of Weights and Measures (BIPM). Quantitative NMR (^1^H-qNMR) was used to characterize primary standards of all veterinary drugs certified. The certified mass fractions of the veterinary drug residues were 490 ± 100 µg/kg for chlorpromazine, 44 ± 4.4 µg/kg for ciprofloxacin, 3.3 ± 1.4 µg/kg for clenbuterol, 9.5 ± 0.8 µg/kg for dexamethasone, 57 ± 4.8 µg/kg for enrofloxacin, 3.0 ± 0.4 µg/kg for meloxicam, 12.4 ± 1.2 µg/kg for ractopamine, and 2290 ± 120 µg/kg for sulfadiazine with expanded uncertainties quoted (95% confidence) which include the effects due to between-bottle inhomogeneity, instability during long-term storage and transportation, and characterization.

**Supplementary Information:**

The online version contains supplementary material available at 10.1007/s00216-023-04794-5.

## Introduction

Veterinary drugs are used on many animal farms to prevent or control diseases, promote growth, increase lean muscle mass, and decrease inflammation or as tranquilizers, among other uses [1, 2]. The residue levels are regulated within Canada and other countries [3–5] and as a result, determination of veterinary drug residues in animal products is important. The presence of residues can lead to potential health risks and create trade barriers [6]. The ability to provide assurance to both domestic consumers and export customers of drug residue concentrations in beef relies on the implementation of true and precise methods of analysis. This assurance is generally improved by laboratory accreditation such as ISO/IEC17025:2017. In addition to proficiency testing schemes, CRMs are often used to demonstrate analyst and/or method competence [7].

Method validation is often conducted by only spiking drug analytes into samples, but oftentimes, the extraction conditions to achieve high recoveries for spiked samples are insufficient to yield acceptable extractability for real samples. Incurred residue is the presence of a chemical in one or more tissues of the body at some time after administration or exposure [8]. This better reflects the true physical, physiochemical, and chemical interactions of the drugs within the animal tissues, and is much preferred to spiked matrix materials where drugs are added to the tissue postmortem. The CRM certification process ensures the correct amount of residue in the tissue has been established by standard methods or by collaborative studies that combine data from several expert laboratories [9]. The certification process also warrants that the CRM is sufficiently homogeneous and stable [9].

For example, a feasibility study for the development of a CRM of nitrofuran metabolites incurred in chicken breast muscle was performed on a small batch with the conclusion that a large batch CRM was feasible [10]. CRMs containing incurred chloramphenicol and nitroimidazoles in pork muscle are available from the European Commission [11, 12] while a CRM containing incurred clenbuterol in mutton muscle is also available [13]. However, CRMs containing multiple classes of incurred veterinary drug residues in muscle tissue were previously unavailable until this work. In this regard, BOTS-1 CRM [14] has been designed and produced to offer a quality control material to be used for calibration and method validation/verification for the analysis of multiple classes of incurred veterinary residues in bovine muscle.

Maximum residue limits (MRLs) and minimum method performance requirements (MMPRs) for veterinary drug residues vary by country [3–5]. While our goal was to produce a bovine muscle CRM containing multiple veterinary drug residues near the Canadian MRLs or European MMPRs, the inherent variability of the biological process in the uptake, distribution, and elimination of the drugs made this very difficult to achieve. Dosage of multiple drugs and selection of an appropriate endpoint was designed and performed in a manner to yield drug residues as near as possible to the MRL or MMPR, while adhering to animal ethics and care guidelines [15]. Processing of the harvested muscle tissue was performed in a manner to preserve the concentration of the drug residues while providing a final product that is stable, easy to work with, and homogeneous.

The analysis of drug residues in animal matrices has been performed using several different techniques including microbiological assays [16, 17], high-performance liquid chromatography (HPLC) [18, 19], gas chromatography–mass spectrometry (GC–MS) [20, 21], liquid chromatography tandem mass spectrometry (LC–MS/MS) [20–24], and liquid chromatography high-resolution mass spectrometry (LC-HRMS) [19, 25–27]. LC–MS methods have become preferred due to their high specificity, sensitivity, scope, and speed (including high-resolution MS detection). External calibration with standards spiked into a blank matrix at the MRL/MMPR or over a calibration range encompassing the MRL/MMPR is the predominant calibration approach for veterinary drug screening [18–27], while standard addition [28–30] and isotope dilution mass spectrometry (ID-MS) [30–32] are used for more accurate analyses.

Independent of the MS analyzer, ID-MS is widely recognized as one of the most accurate approaches to quantitative analysis [33, 34]. Despite its accuracy, it is not routinely used for screening or confirming veterinary drug residue levels due to the perceived complexity of the calibration approach and the requirement to estimate preliminary concentrations of drug residues for each sample. This is a time-consuming process which does not lend itself to the analysis of a large number of samples with widely varying concentrations. However, ID-MS is useful in the production of certified reference materials [30–33, 35] where accuracy, not throughput, is the main concern.

**Fig. 1:**
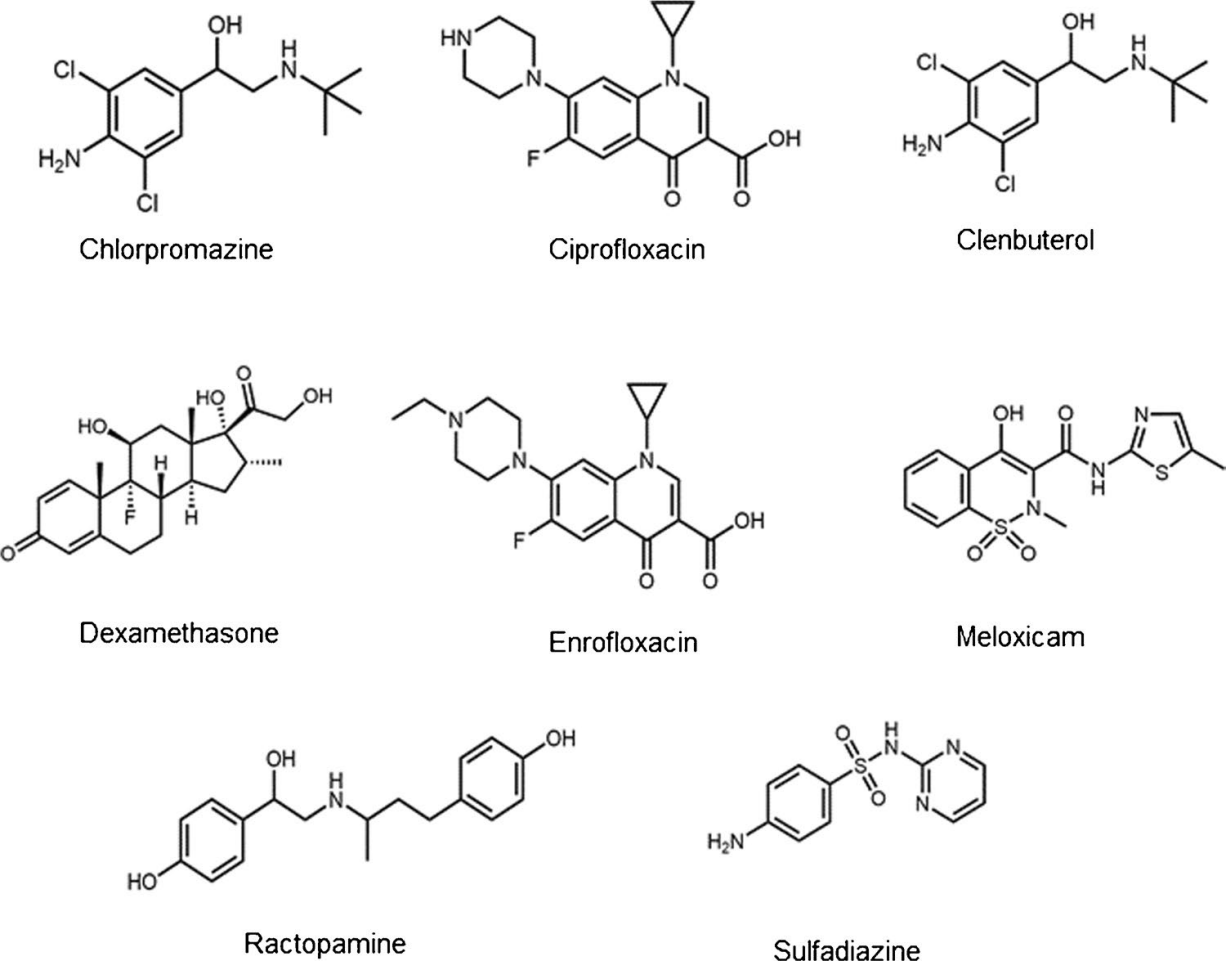
Chemical structures of veterinary drugs certified in BOTS-1 CRM

In this report, we will highlight the production and certification of BOTS-1 for a subset of the dosed drugs and metabolite residues, including chlorpromazine (Cprom), ciprofloxacin (Cipro), clenbuterol (Clen), dexamethasone (Dexa), enrofloxacin (Enro), meloxicam (Meloxi), ractopamine (Racto), and sulfadiazine (Sulfa) (Fig. 1) in freeze-dried bovine muscle tissue using multi-residue LC–MS/MS methods coupled with double isotope dilution mass spectrometry (ID^2^MS) and standard addition–double isotope dilution mass spectrometry (SA-ID^2^MS) techniques [36]. Assessment of sample homogeneity and stability will be described, along with consensus value assignment from results generated in collaborating laboratories. Other drug residues present in BOTS-1 have not yet been certified).

## Experimental

### Chemicals and reagents

Materials for primary reference standards for Cipro, Cprom HCl, Dexa, Enro, Meloxi, and Sulfa were purchased from Sigma-Aldrich (Oakville ON, Canada), Clen HCl was purchased from Toronto Research Chemicals (Toronto ON, Canada), and Racto HCl was purchased from LKT Laboratories (St. Paul, MN, USA). Stable isotope labeled standards for [^2^H_3_]-Meloxi and [^13^C_6_]-Sulfa were purchased from Sigma-Aldrich (Oakville ON, Canada), [^13^C_1_,^2^H_3_]-Cprom standard was purchased from Toronto Research Chemicals (Toronto ON, Canada), and standards for [^2^H_8_]-Cipro HCl, [^2^H_9_]-Clen, [^2^H_4_]-Dexa, [^2^H_5_]-Enro HI, and [^2^H_6_]-Racto HCl were purchased from CDN (Pointe-Claire QC, Canada). Maleic acid and dimethyl sulfone were purchased from Fluka (Oakville ON, Canada), 3,5-dinitrobenzoic acid was obtained from Aldrich (St. Louis MO, USA), and benzoic acid SRM 350b standard was purchased from the National Institute of Standards and Technology (NIST; Gaithersburg, MD, USA).

High-purity deionized water was collected from a Millipore Milli-Q Advantage A10 mixed bed ion exchange system fed with reverse osmosis domestic water (Jaffrey, NH, USA). Optima LC/MS grade acetonitrile and methanol, formic acid, ascorbic acid, and HPLC grade isopropanol were purchased from Fisher Scientific (Fair Lawn, NJ, USA). Hexane (95% *n*-hexane) was purchased from Caledon Laboratories (Georgetown, ON, USA), and NH_4_OH (30% solution) was purchased from EMD Chemicals (Gibbstown, NJ, USA).

### Preparation of BOTS-1

BOTS-1 was produced in a two-stage process. The first stage involved a selection of suitable pharmaceuticals, selection of a single, healthy, market-age veterinary-inspected heifer, administration of the drugs to the heifer, euthanasia, harvest of the muscle tissue, and grinding to a hamburger-like consistency. The veterinary drugs were selected to include appropriate therapeutic classes and were based on their registration for livestock use, existence of MRLs for allowed substances, or MMPRs for banned substances (Table S-1, Supplemental Material). Metabolites of dosed drugs were also expected to be found post-euthanization. Ethical and practical concerns of animal welfare and health were addressed by following the Guide to the Care and Use of Experimental Animals [15]. The therapeutic dose levels, dose timing, and routes of administration were selected based on known pharmacokinetic properties and a target range equal to the MRL or MMPR for the incurred residue concentrations. A healthy bovine heifer was fed a diet that was in compliance with the Health of Animals Regulations [37] and was subjected to the Supply Animal and Use Protocol (pharmaceutical dosing) supplied by the Canadian Food Inspection Agency. The heifer was individually shipped for slaughter at a facility approved by the Domestic Meat Inspection Program (Saskatchewan Ministry of Agriculture). After euthanasia, several organs and the muscle tissue were harvested. No neck muscle was collected to avoid muscle near the injection sites and visible fat was trimmed as much as possible. Approximately 120 kg of muscle was collected. The meat was double ground, packaged into plastic sausage casings, and stored at − 80 °C.

The second stage of BOTS-1 production involved the preparation of lyophilized muscle tissue by wet homogenization, freeze-drying, milling to a fine powder-like consistency, dry homogenization, and bottling in 10 g aliquots. The beef sausages (122 kg total mass) were allowed to thaw before the casings were removed. Wet milling was performed with a 40 L Stephan Universal Machine (1000–2500 rpm for 15 min with a small amount of ice added to keep the temperature low). Freeze-drying was performed under a vacuum (0.25 mmHg) with a condenser temperature of − 55 °C. The freeze-dried beef cakes were stored at 2–4 °C until dry-grinding was performed using a Stephan Universal Machine (1200 rpm until the temperature rose to 21 °C). The bulk freeze-dried, ground beef (39 kg) was then transferred to a 180 L polyethylene mixing drum, purged with argon, and mixed for 9 h. Aliquots (10 g) were manually transferred into 60 mL amber wide-mouth glass bottles, flushed with argon, and sealed with Teflon-lined polyethylene caps. In total, over 3400 bottles of BOTS-1 were produced and stored at − 80 °C at the NRC.

## Methods

### Purity of primary reference standards by qNMR

Chemical purity of the primary reference standards was established using ^1^H-qNMR (Avance-III 400 MHz NMR spectrometer, Bruker) using general methods described previously [38]. Samples were prepared gravimetrically in triplicate to contain both the primary reference standard and an internal standard. Purity of all internal standards is traceable to the International System of Units (SI) through NIST SRM 350b benzoic acid standard from previous qNMR value assignment at NRC. The purity (mass fraction) of Cipro, Clen, Enro, Meloxi, and Racto was determined with ^1^H-qNMR using maleic acid as an internal standard, whereas 3,5-dinitrobenzoic acid internal standard was employed for Cprom HCl and Dexa, and dimethyl sulfone served as the internal standard for Sulfa.

### Isotope dilution techniques

**Fig. 2:**
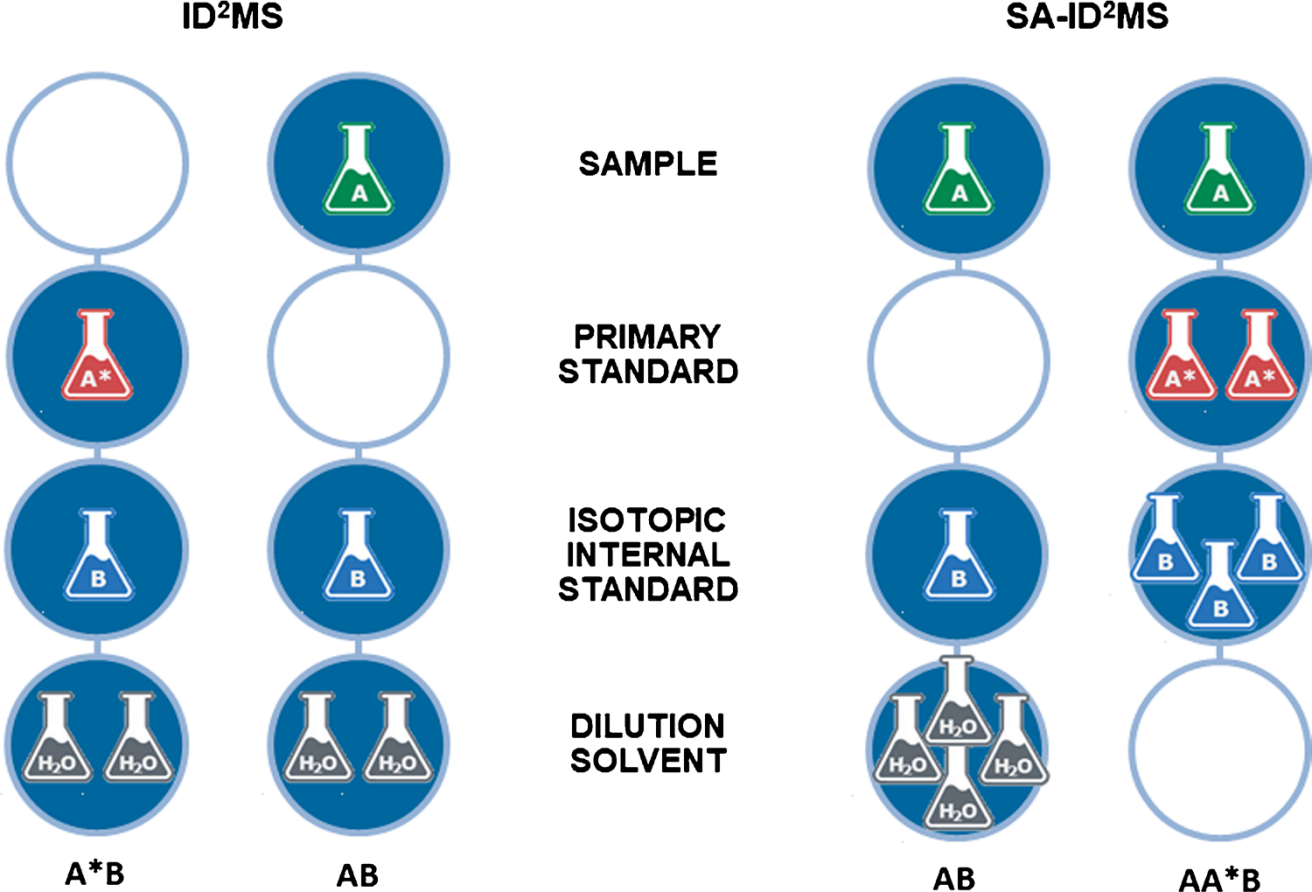
Conceptual scheme of implementing ID^2^MS and SA-ID^2^MS indicating components added to each sample mixture and their relative proportions; A = analyte in the sample (natural isotopic composition); A^*^ = analyte in the primary standard (natural isotopic composition); B = analyte in the isotopic standard (isotopically enriched)

For the analysis of veterinary drug residues in BOTS-1, initial estimates of mass fractions were first determined using the methods described herein in an iterative manner. Once confidence was established in the estimated mass fractions, two ID-MS approaches were applied. The first approach, exact matching double isotope dilution mass spectrometry [34, 39] (ID^2^MS, Fig. 2 and Eq. 1) required two sample mixtures: a matrix sample spiked with a stable label internal standard at a concentration which provides a 1:1 peak area ratio of the drug and the internal standard and a calibration solution prepared in solvent using the same internal standard (B) and a primary standard (A*) of the drug (A) such that the peak area ratio is also near 1:1. This approach utilizes the following measurement model:

1 $${w}_{\mathrm{A}}={w}_{{\mathrm{A}}^{*}}\frac{{m}_{{\mathrm{A}}^{*}({\mathrm{A}}^{*}\mathrm{B})}{m}_{\mathrm{B}(\mathrm{AB})}\left({R}_{{\mathrm{A}}^{*}\mathrm{B}}-{R}_{\mathrm{A}*}\right)\left({{R}_{\mathrm{AB}}-R}_{\mathrm{B}}\right)}{{m}_{\mathrm{A}(\mathrm{AB})}{m}_{\mathrm{B}({\mathrm{A}}^{*}\mathrm{B})}\left({R}_{\mathrm{B}}-{R}_{{\mathrm{A}}^{*}\mathrm{B}}\right)\left({R}_{\mathrm{A}}-{R}_{\mathrm{AB}}\right)}$$

where:

A: analyte in the sample (natural isotopic composition)
A^*^: analyte in the primary standard (natural isotopic composition)
B: analyte in the isotopic standard (isotopically enriched)
*w*_A_: mass fraction of A in sample
*w*_A*_: mass fraction of A* in the primary standard
*m*_X(XY)_: mass of the component X in blend of X and Y
*R*_X_: peak area ratio of A/B in the blend X

The second approach was exact matching standard addition–double isotope dilution mass spectrometry (SA-ID^2^MS, Fig. 2 and Eq. 2), which combined standard addition with ID^2^MS [36, 40]. This approach also required two sample mixtures: a matrix sample spiked with stable label internal standard at a concentration which provides a 1:1 peak area ratio of the drug and the internal standard after extraction, as in the ID^2^MS approach, and a second matrix sample spiked with both drug and internal standard such that the peak area ratio of the drug and the internal standard is also 1:1. For analysis of BOTS-1, the drug was spiked at two times the incurred concentration and the internal standard at three times the drug concentration to provide a 1:1 ratio after extraction. Since this approach used two samples, both extracted from the matrix, the potential effects of the sample matrix were taken into account. This approach utilizes the following measurement model:

2 $${w}_{\mathrm{A}}={w}_{{\mathrm{A}}^{*}}\frac{{m}_{\mathrm{A}*(\mathrm{AA}*\mathrm{B})}{m}_{\mathrm{B}\left(\mathrm{AB}\right)}\left({R}_{\mathrm{AB}}-{R}_{\mathrm{B}}\right)\left({R}_{{\mathrm{A}}^{*}}-{R}_{\mathrm{AA}*\mathrm{B}}\right)}{\begin{array}{c}+{m}_{\mathrm{A}\left(\mathrm{AB}\right)}{m}_{\mathrm{B}\left(\mathrm{AA}*\mathrm{B}\right)}\left({R}_{\mathrm{AB}}-{R}_{\mathrm{A}}\right)\left({R}_{\mathrm{B}}-{R}_{\mathrm{AA}*\mathrm{B}}\right)\\ +{m}_{\mathrm{A}(\mathrm{AA}*\mathrm{B})}{m}_{\mathrm{B}\left(\mathrm{AB}\right)}\left({R}_{\mathrm{B}}-{R}_{\mathrm{AB}}\right)\left({R}_{\mathrm{A}}-{R}_{\mathrm{AA}*\mathrm{B}}\right)\end{array}}$$

Natural working solutions (NWS) containing the drug analyte standards and internal standard working solutions (ISWS) containing the stable isotope internal standards were prepared at concentrations approximately six times the expected concentration of the analytes in the matrix such that a 100 µL spike of ISWS into a 0.5 g sample of BOTS-1 (mix AB) was devised to provide a 1:1 peak area ratio for the natural and isotopically labeled analyte signals while a 300 µL spike of ISWS and a 200 µL spike of NWS into a 0.5 g sample of BOTS-1 (mix AA*B) should have also provided a 1:1 peak area ratio. NWS-1 was prepared for method multi-residue-1 (MR-1) and NWS-2 was prepared for method multi-residue-2 (MR-2).

NWS-1 containing Cipro, Clen, Enro, and Racto was prepared in a 50:50 mixture of methanol and water while NWS-1B for Sulfa was prepared in an 85:15 mixture of 10 mM HCl and acetonitrile. ISWS-1A containing [^2^H_8_]-Cipro, [^2^H_9_]-Clen, [^2^H_5_]-Enro, and [^2^H_6_]-Racto was prepared in a 50:50 mixture of methanol and water while ISWS-1B containing [^13^C_6_]-Sulfa was prepared in an 85:15 mixture of 10 mM HCl and acetonitrile. The calibration solution (mix A*B) for Cipro, Clen, Enro, and Racto was prepared in a 100:900:1 mixture of methanol, water, and formic acid, while the CAL solution for Sulfa was prepared in a 95:5 mixture of acetonitrile and 10 mM HCl.

NWS-2 containing Cprom, Dexa, and Meloxi and ISWS-2 containing [^13^C_1_,^2^H_3_]-Cprom, [^2^H_4_]-Dexa, and [^2^H_3_]-Meloxi were prepared in a 50:50 mixture of methanol and water. The calibration solutions were prepared in a 600:400:1 mixture of acetonitrile, water, and formic acid containing 4 mM ascorbic acid.

### Extraction methods

A universal extraction procedure was determined to be insufficient for CRM certification given the structural variety of the analytes. Therefore, several different extraction procedures were employed to ensure maximum recovery of all veterinary drugs.

#### Multi-residue-1A (MR-1A): Cipro, Clen, Enro, Racto

Cipro, Clen, Enro, and Racto were extracted from BOTS-1 CRM via liquid–solid extraction. After reconstitution of 0.5 g of BOTS-1 with 1 mL of water and gravimetric addition of internal standard, the sample was extracted with 4 mL of an 80:10:10 mixture of acetonitrile, isopropanol, and water via shaking for 30 min. The sample was centrifuged and the supernatant retained. The extraction was repeated and the supernatants were combined. Defatting was performed by adding 2 mL of hexane to the combined supernatants and shaking for 5 min. The sample was centrifuged to separate the phases and the hexane was removed and discarded. An aliquot (4 mL) of the sample extract was concentrated under vacuum to 450 µL followed by the addition of 50 µL of MeOH. All samples were filtered through 0.2 µm PTFE filter vials prior to injection onto the LC–MS system. The equivalent sample concentration was 0.44 g/mL in MR-1A final extracts.

#### Multi-residue-1B (MR-1B): Sulfa

After reconstitution of 0.5 g of BOTS-1 with 1.0 mL of water and gravimetric addition of internal standard, the sample was extracted with 10 mL of 100 mM HCl in acetonitrile via shaking for 30 min. The sample was centrifuged and the supernatant retained. The extraction was repeated with 10 mL of 10 mM HCl in acetonitrile and the supernatants were combined. Defatting was performed by adding 5 mL of hexane to the combined supernatants and shaking for 5 min. The sample was centrifuged to separate the phases and the hexane was removed and discarded. An aliquot of the remaining supernatant (250 µL) was diluted two-fold with 10 mM HCl. All samples were filtered through 0.2 µm PTFE filter vials prior to injection onto the LC–MS system. The equivalent sample concentration was 0.012 g/mL in MR-1B final extracts.

#### Multi-residue-2: Cprom, Dexa, Meloxi

Cprom, Dexa, and Meloxi were extracted from BOTS-1 CRM via liquid–solid extraction. After reconstitution of 0.5 g of BOTS-1 with 1 mL of water, 20 µL of 1 M ascorbic acid was added followed by gravimetric addition of internal standard. The samples were extracted with 4 mL of acetonitrile via shaking for 20 min. The samples were centrifuged and the supernatant retained. The extraction was repeated with the supernatants combined followed by concentration under vacuum to 100 µL. Step-wise reconstitution with 0.1% formic acid in acetonitrile (200 µL), 20 mM ascorbic acid (100 µL), and water (100 µL) was performed. The samples were placed at 4 °C for 30 min, followed by centrifugation to remove precipitates. All samples were filtered through 0.2 µm PTFE filter vials prior to injection onto the LC–MS system. The equivalent sample concentration was 0.96 g/mL in MR-2 final extracts.

### LC–MS/MS methods

#### MR-1A: Cipro, Clen, Enro, Racto

A 4 µL sample aliquot was injected onto an LC–MS/MS system consisting of a 1290 Infinity I UPLC (Agilent Technologies; Mississauga, ON, Canada) interfaced to a TSQ Quantiva triple quadrupole mass spectrometer equipped with electrospray ionization (Thermo Fisher Scientific, San Jose, CA, USA). Separation was carried out with mobile phases consisting of water and formic acid (1000:1, A) and acetonitrile with formic acid (1000:1, B) on an Ace-3 C18 column (50 mm × 2.1 mm, 3 µm; Advanced Chromatography Technologies, Aberdeen, Scotland) with integrated guard column and column heater set to 40 °C. A gradient mobile phase flow of 0.4 mL/min with 5% B (0–1.0 min), 5 to 25% B (1.0–4.0 min), followed by a column wash with 98% B and equilibration back to 5% B was used for separation. The total run time was 6.5 min. The MS system was operated with electrospray ionization in positive ion mode with ion spray voltage set to 4000 V, sheath gas at 55, aux gas set to 20, and sweep gas set to 2 arbitrary units. The ion transfer tube was set to 325 °C and vaporizer temperature set to 280 °C. Argon was used as collision gas at 1.5 mTorr and Q1/Q3 resolution was set to 0.7.

#### MR-1B: Sulfa

A 2 µL sample aliquot was injected onto the same LC–MS/MS system, column and mobile phases used in MR-1A. A gradient mobile phase flow of 0.4 mL/min with 2% B (0–1.0 min), 2 to 30% B (1.0–2.5 min), followed by a column wash with 98% B and equilibration back to 2% B was used for separation. The total run time was 5.0 min. The MS system was operated under the same conditions as method MR-1A.

#### MR-2A: Cprom and Meloxi

A 10 µL sample aliquot was injected onto the same LC–MS/MS system, column and mobile phases used in MR-1A. A gradient mobile phase flow of 0.4 mL/min with 30% B (0–1.0 min), 30 to 40% B (1.0–5.0 min), followed by a column wash with 98% B and equilibration back to 30% B was used for separation. The total run time was 8.0 min. The MS system was operated under the same conditions as method MR-1A.

#### MR-2B: Dexa

A 10 µL sample aliquot was injected onto the same LC–MS/MS system, column and mobile phases used in MR-1A. A gradient mobile phase flow of 0.4 mL/min with 20% B (0–1.0 min), 20 to 40% B (1.0–4.0 min), followed by a column wash with 98% B and equilibration back to 20% B was used for separation. The total run time was 7.0 min. The MS system was operated with ESI in negative ion mode with all other conditions the same as method MR-1A. The dexamethasone formate adduct was monitored in quadrupole 1 (Table 1).

**Table 1:** Mass spectrometer acquisition parameters for the veterinary drug residues

Method	Analyte	Ionization polarity	Ion transitions (*m/z*)	CE (eV)
MR-1A	Cipro	+	332.2 → 205.2/245.2	31/24
	Clen	+	277.2 → 203.1/132.1	16/29
	Enro	+	360.2 → 316.2/245.2	18/27
	Racto	+	302.2 → 107.2/121.2	32/23
MR-1B	Sulfa	+	251.2 → 156.1/108.1	18/27
MR-2A	Cprom	+	319.2 → 214.0/210.0	44/55
	Meloxi	+	325.2 → 115.1/141.1	20/21
MR-2B	Dexa	–	437.2 → 361.2/307.2	− 20/ − 35

## Results and discussion

### Quantitative ^1^H-qNMR

^1^H-qNMR was used to assign mass fractions to the veterinary drug primary standards. This technique is a primary analytical method that allows compound-independent calibration to determine the mass fraction of the analyte using an appropriate internal standard [32, 41–43]. The results are traceable to the International System of Units (SI) through gravimetric preparation of standards of established purity on calibrated balances using traceable mass standards. The qNMR results for the veterinary drug primary reference standards are shown in Table 2.

**Table 2:** ^1^H-qNMR mass fraction assignment (with internal standard used for calibration) for veterinary drug standards. Expanded uncertainties are quoted (95% confidence)

Analyte	Mass fraction (mg/g)	Primary calibrator
Cprom HCl	996.6 ± 3.6	3,5-dinitrobenzoic acid
Cipro	997.0 ± 4.7	Maleic acid
Clen HCl	980.6 ± 3.7	Maleic acid
Dexa	999.4 ± 5.3	3,5-dinitrobenzoic acid
Enro	997.7 ± 9.4	Maleic acid
Meloxi	995.6 ± 9.9	Maleic acid
Racto	960.3 ± 2.8	Maleic acid
Sulfa	996.9 ± 3.5	Dimethyl sulfone

### Extraction

Several aspects for the extraction of the veterinary drug residues from BOTS-1 were evaluated, including the effect of solvent composition, shaking time, number of serial extractions, sample cleanup, and sample equilibration time after spiking. All BOTS-1 samples were accurately weighed and re-hydrated with two parts water prior to extraction to ensure equivalency of hydration with actual study samples. Analytes were extracted in two groups largely based on their polarity.

#### Multi-residue-1: Cipro, Clen, Enro, Racto, and Sulfa

Solvent compositions similar to those found in the literature were evaluated for recovery of the measurands [18, 24, 44–47]. Similar recoveries were observed with acetonitrile and methanol, with or without water or formic acid. An 80:10:10 solvent mixture of acetonitrile, isopropanol, and water was chosen for Cipro, Clen, Enro, and Racto. An extraction solvent consisting of acetonitrile and hydrochloric acid showed slightly higher recoveries for Sulfa and was used for Sulfa mass fraction assignment. A shaking time of 30 min was found to be optimal (Fig. S-1, Supplemental Material) with consistent analyte/internal standard peak area ratios (Fig. S-2, Supplemental Material). Serial extractions (1 to 4) were carried out to assess recovery and potential changes in the analyte/internal standard peak area ratio. The results indicated that four extractions provided near complete recovery; however, two extractions provided ≥ 90% recovery of all analytes with consistent analyte/internal standard peak area ratios (Fig. S-3, Supplemental Material). A hexane wash of the supernatant was evaluated as a further cleanup step to remove lipids and other non-polar endogenous components and was found to provide a visually cleaner sample with no effect on analyte recovery. Sample equilibration time after spiking internal standard and/or analyte into re-hydrated BOTS-1 was evaluated with no differences observed over a 16 h period.

#### Multi-residue-2: Cprom, Dexa, and Meloxi

Solvent compositions similar to those found in the literature were evaluated for recovery of these analytes [24, 44, 45, 48]. Similar recoveries were observed with acetonitrile or methanol; however, no recovery of Cprom was observed with acidified solvents. pH control of the extraction was evaluated with the addition of buffers to the re-hydrated samples prior to spiking and extraction. The anti-oxidant, ascorbic acid (1 M, 20 µL), was found to increase recovery of Cprom significantly but had minimal effects on Dexa or Meloxi. A final extraction solvent composition of acetonitrile was chosen with prior addition of ascorbic acid. A shaking time of 20 min was found to be optimal (Fig. S-4, Supplemental Material) with consistent analyte/internal standard peak area ratios (Fig. S-5, Supplemental Material). Results of serial extractions indicated that four extractions provided near complete recovery; however, two extractions provided ≥ 95% recovery of all analytes with consistent analyte/internal standard peak area ratios (Fig. S-6, Supplemental Material). A flash freeze step was evaluated as a further cleanup step to remove lipids and other non-polar endogenous components; however, significant analyte losses were observed. Sample equilibration time after spiking internal standard and/or analyte into re-hydrated BOTS-1 was evaluated with no differences observed over a 16 h period.

### LC–MS/MS detection

**Fig. 3:**
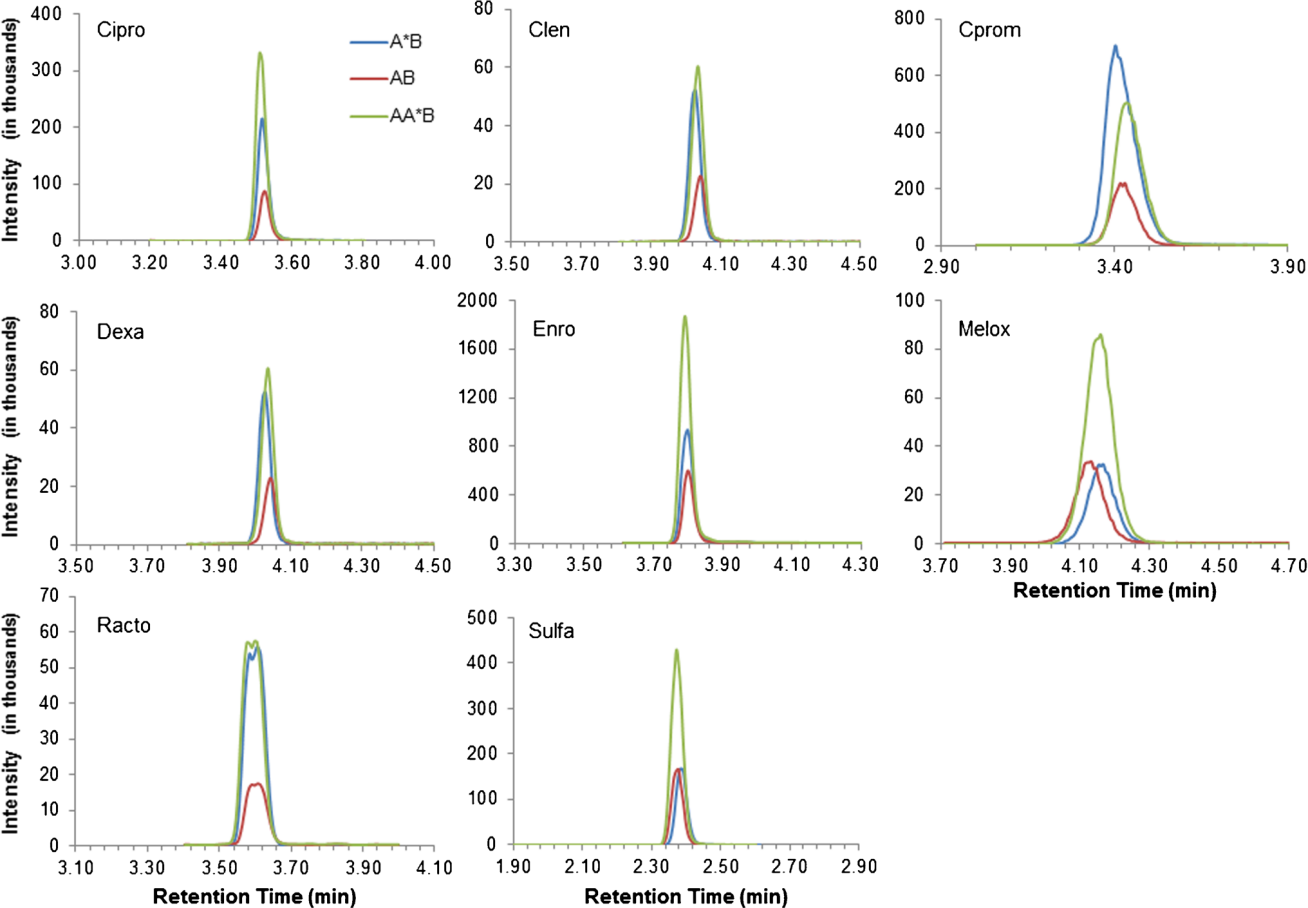
Chromatograms of veterinary drug residues from CAL standard solution (A*B), ID^2^MS sample extracted from BOTS-1 (AB), and SA-ID^2^MS sample extracted from BOTS-1 (AA*B). Proportions of sample, primary standard, and isotopic internal standard are shown in Fig. 2 and should provide a peak area ratio of 1:1:3 in the absence of ion suppression (given complete analyte recovery), as observed for Sulfa and Meloxi

The samples were prepared as described in the isotope dilution techniques and extraction sections and injected onto the LC–MS/MS system with the parameters described above in LC–MS/MS methods. For each analyte, peak areas from two different product ions were determined with results calculated independently and averaged afterwards. In all cases, the results from the two different product ions provided similar results, demonstrating consistent ion ratios free of matrix interferences. Representative LC–MS/MS chromatograms of the most sensitive ion transitions for each analyte from a CAL (mix A*B), BOTS-1 ID^2^MS sample (mix AB), and BOTS-1 SA-ID^2^-MS sample (mix AA*B) are shown in Fig. 3.

### Matrix effect

Matrix effect, a percentage difference of response for an equivalent concentration of the analyte in the final matrix extract vs. reagent-only solution, was evaluated for all analytes using the labeled internal standards as surrogates. The ISs were spiked into CAL and BOTS-1, processed, and run by LC–MS/MS. A value of zero indicates no matrix effect, a value > zero indicates ion suppression, and a value < zero indicates ion enhancement. Cprom exhibited the highest amount of ion suppression with Dexa, Racto, and Clen also exhibiting high signal suppression (Table 3). With the exception of clenbuterol, we have observed positive trends between the extent of ion suppression and the relative difference between ID^2^-MS and SA-ID^2^-MS results (Fig. S-7, Supplemental Material). Since all analytes have a co-eluting stable isotope internal standard for quantification, ion suppression or enhancement is largely normalized with minimal effects on quantitation yet we are able to see minor unaccounted effects by comparing the results of ID^2^MS and SA-ID^2^MS results).

**Table 3:** Matrix effect

Analyte	Matrix effect (%)
Cprom	73
Cipro	33
Clen	45
Dexa	63
Enro	9
Meloxi	29
Racto	52
Sulfa	0

### Homogeneity

Between-bottle homogeneity of the drug residue levels in BOTS-1 was assessed using duplicate sub-samples (0.5 g) taken from 15 randomly selected bottles over the full range of the batch of 3418 bottles. All samples were analyzed via LC–MS/MS in a single batch [49]. Results are shown in Figures S-8 to S-15 (Supplemental Material) with error bars representing the standard uncertainties of duplicate sample results. Enro and Sulfa were extracted using method MR-1A with Cipro, Clen, and Racto as this method provided acceptable precision for all analytes. Since the measurement uncertainty exceeded the uncertainty due to homogeneity (*u*_hom_), a random effects statistical model was used to separate these uncertainty contributions. We employed the DerSimonian-Laird (DSL) estimator [50] as implemented in the R package metafor [51]. This model was fitted to the 15 × 2 experimental data and the confidence intervals of the model parameters (which include the uncertainty due to inhomogeneity) were estimated using the R function ‘confint’.

With the sole exception for chlorpromazine, the DSL method failed to provide an estimate of homogeneity (i.e., *u*_hom_ = 0). To address this concern, we adopted the upper 95% confidence bound, *u*_hom_ + 2*u*(*u*_hom_), as the estimate for homogeneity [49]. This approach yielded the maximum conservative estimates for Cipro, Clen, Meloxi, Racto, and Sulfa, however still provided a *u*_hom_ estimate of zero for Enro and Dexa. For Enro and Dexa, *u*_hom_ was therefore estimated at 0.8% based on the uncertainty of the other analytes. Chlorpromazine showed a slight downward trend in mass fraction over the batch; however, this trend could not be attributed solely to the matrix material or the method and was therefore reported as a homogeneity uncertainty.

The within-bottle homogeneity of the veterinary drug residue levels in BOTS-1 was assessed using five sub-samples (0.5 g) from the same bottle. The results were evaluated using the DerSimonian-Laird random effects model [50]. The uncertainty component for BOTS-1 due to within-bottle homogeneity was combined with the between-bottle homogeneity component and reported as a combined homogeneity component.

The combined calculated relative uncertainty components for BOTS-1 due to homogeneity were 5.7% for Cprom, 0.9% for Cipro, 0.9% for Clen, 0.8% for Dexa, 0.8% for Enro, 1.7% for Meloxi, 1.0% for Racto, and 0.5% for Sulfa.

### Stability

BOTS-1 was processed, bottled, and stored under argon at − 80 °C. Three types of stability were evaluated for the veterinary drug residues in BOTS-1: short-term transportation (ST), freeze–thaw (FT), and long-term storage (LT) stability [49]. The individual uncertainties for each stability type were combined and reported as a single stability component (*u*_stab_) as shown in Table 4.

**Table 4:** Value assignment and uncertainty budget for veterinary drug residues in BOTS-1 (see text for definitions)

Analyte	*w*_A_ (ng/g)	*U*_c, 95%_ (ng/g)	*u*_c_ (ng/g)	*u*_char_ (ng/g)	*u*_hom_ (ng/g)	*u*_stab_ (ng/g)
Cprom	490	100	50	36	26	26
Cipro	44	4.4	2.2	2.0	0.42	0.78
Clen	3.3	1.4	0.7	0.7	0.03	0.0
Dexa	9.5	0.8	0.4	0.4	0.07	0.0
Enro	57	4.8	2.4	1.2	0.4	2.0
Meloxi	3.0	0.4	0.2	0.2	0.05	0.0
Racto	12.4	1.2	0.6	0.6	0.13	0.0
Sulfa	2290	120	60	25	10	53

A short-term (ST) stability evaluation was performed with samples from three different bottles for each temperature. Bottles were stored at − 80 °C, − 20 °C, 6 °C, 20 °C, and 37 °C for two weeks with − 80 °C serving as the control temperature. Sub-samples were removed after 2 weeks and stored at − 80 °C until all samples were analyzed via LC–MS/MS in a single batch for Cipro, Clen, Enro, Racto, and Sulfa and a separate single batch for Cprom, Dexa and Meloxi. Results are shown in Figures S-16 and S-17 (Supplemental Material) as mass fractions relative to the − 80 °C control samples versus storage temperature, with error bars representing the standard deviation of replicate sample results. Analysis of the results by DSL and linear regression analysis indicated that the samples stored at − 20 °C, 6 °C, 20 °C, and 37 °C are in agreement with the samples stored at − 80 °C for all analytes except for Cprom. Upon further evaluation of the Cprom results, the mass fraction of the 6 °C samples and the 37 °C samples were low relative to the − 80 °C sample, and a short-term stability uncertainty component (DSL) was included in the combined stability uncertainty.

A freeze–thaw (FT) stability evaluation was performed using a single bottle which was subjected to 20 FT cycles. Duplicate sub-samples were taken at 1, 5, 10, 15, and 20 freeze–thaw cycles. All samples were stored at − 80 °C and were analyzed via LC–MS/MS in a single batch for Cipro, Clen, Enro, Racto, and Sulfa and a separate single batch for Cprom, Dexa, and Meloxi. Results are shown in Figures S-18 and S-19 (Supplemental Material) as mass fractions relative to one freeze–thaw cycle versus the number of freeze–thaw cycles, with error bars representing the standard deviation of replicate sample results. Analysis of the results by DSL and linear regression analysis indicated that the results, up to 20 FT cycles, are in agreement with the comparison sample (0 FT cycles) for all measurands. As a result of the observed freeze–thaw stability results, no uncertainty components were included in the combined uncertainties.

A long-term stability (LT) evaluation was performed using three different bottles for each temperature stored at − 80 °C. Sub-samples were removed at fourteen (14), twenty-seven (27), and seventy-one (71) months and analyzed via LC–MS/MS for Cipro, Clen, Enro, Racto, and Sulfa. Sub-samples were removed at seven (7), sixteen (16), and sixty-one (61) months and analyzed in a single batch for Cprom, Dexa, and Meloxi. Results are shown in Figures S-20 and S-21 (Supplemental Material) as mass fractions relative to time zero versus storage time, with error bars representing the standard deviation of replicate sample results. Stability analyses were performed with a fresh preparation of all reference solutions from solid reference standards. Analysis of the results by DSL and linear regression analysis indicated that the samples stored at − 80 °C are in agreement with the time zero values for Clen, Dexa, Meloxi, and Racto while effects were observed for Cprom, Cipro, Enro, and Sulfa. Chlorpromazine showed an upward trend in mass fraction over time; however, this trend could not be attributed solely to the matrix material or the method. As a result of the observed long-term stability results, uncertainty components were included in the combined stability uncertainty for Cprom, Cipro, Enro, and Sulfa.

### External laboratory data

BOTS-1 was analyzed by three external laboratories, CFIA, USDA, and BVL, each using their own multi-residue LC–MS/MS methods, and by 13 additional laboratories participating in the key comparison study CCQM K141/P178 [14]. CCQM K141/P178 included results for Enro and Sulfa. External laboratory data is shown in Table S-2 (Supplemental Material).

### Value assignment and combined uncertainty

Certified mass fraction values were assigned for Cprom, Cipro, Clen, Dexa, Enro, Meloxi, Racto, and Sulfa in BOTS-1 by combining all relevant data from NRC, CFIA, USDA, BVL, and CCQM K141/P178 [14] as shown in Table S-2 (Supplemental Material). For each analyte, a Bayesian Gaussian Random Effects Model was used to establish the certified mass fractions [38].

Uncertainty due to characterization was derived via an estimation of the dark uncertainty (overdispersion) from inter-laboratory comparison study CCQM-K141/P178 data for Enro and Sulfa. The dark uncertainty was estimated using the DerSimonian-Laird method [50] and was adopted to other analytes using a Horwitz-type function which models measurement uncertainties in a power-law dependence of the mass fraction of analytes [52, 53]. Consensus values were obtained using a Gaussian random effects model using the data whereby the NRC uncertainty results were expanded using the estimates obtained from the Horowitz-type function:

3 $$u\mathrm{r}\left(t\right)=a{(w/(\mathrm{ng}/\mathrm{g}))}^{-0.15}$$

where *u*_r_(*t*) is the relative dark uncertainty and coefficient *a* was estimated from the values of *u*_r_(*t*) for Sulfa and Enro obtained from the inter-laboratory study (*a* = 0.086)

The overall combined uncertainty (*u*_c_), calculated in accordance with GUM [54], as shown in Eq. 4, includes uncertainties related to within- and between-bottle inhomogeneity (*u*_hom_), uncertainties related to instability (*u*_stab_), and uncertainties related to characterization (*u*_char_). The characterization uncertainty included contributions from measurement, primary standards, and the additional dark uncertainty component revealed from the analysis of the CCQM-K141/P178 data. The certified mass fraction values (*w*_A_) and uncertainties are shown in Table 4.

4 $${u}_{\mathrm{c}}=\sqrt{{u}_{\mathrm{hom}}^{2}+{u}_{\mathrm{stab}}^{2}+{u}_{\mathrm{char}}^{2}}$$

## Conclusions

The preparation and certification of BOTS-1, a unique bovine muscle CRM containing multiple incurred veterinary drug residues, has been presented. The material has been shown to be homogeneous and stable during transportation, multiple samplings, and long-term storage for certified analytes. Isotope dilution methods used in the certification process were described and inter-laboratory data were contributed to the value assignment process. BOTS-1 is intended to be used as a quality control material for calibration and method verification and will contribute to standardization and improvement of measurement capability with SI traceability for veterinary drug analysis.

Relative to a biological tissue reference material that has been spiked with known levels of desired contaminants, this naturally incurred material should provide a truer test of method performance. As some drug residues are known to be predominantly bound to proteins or lipids, a spiked reference material could require less harsh extractions conditions, and if used for method development and validation could lead to under-reporting of residue levels in real test samples. Therefore, the naturally incurred certified reference material described above offers the challenges of a real-world sample and will be a useful tool for food testing laboratories striving to achieve accurate results.

### Supplementary Information

Below is the link to the electronic supplementary material.

Supplementary file1 (DOCX 462 KB)

## References

[1] Melendez DM, Marti S, Pajor EA, Sidhu PK, Gellatly D, Janzen ED, et al. Pharmacokinetics of oral and subcutaneous meloxicam: effect on indicators of pain and inflammation after knife castration in weaned beef calves. Plos One. 2019;14(5). 10.1371/journal.pone.0217518 PMC6534336 31125384

[2] Bryant TC, Engle TE, Galyean ML, Wagner JJ, Tatum JD, Anthony RV (2010). Effects of ractopamine and trenbolone acetate implants with or without estradiol on growth performance, carcass characteristics, adipogenic activity, and blood metablites in feedlot steers and heifers. J Anim Sci.

[3] Health Canada, Veterinary Drugs, Maximum Residue limits (MRLs) 2018 [Available from: https://www.Canada.ca/en/health-canada/services/drugs-health-products/veterinary-drugs/maximum-residue-limits-mrls.html. Accessed 22 Dec 2022

[4] Title 21 of the Code of Federal Regulation (CFR), part 556: Appendix IV. US Residue Limits for Veterinary Drugs, Food Additives, and Unavoidable Contaminants in Meat, Poultry, and Egg Products. 2001.

[5] EU Commission Regulation No 37/2010 on pharmacologically active substances and their classification regarding maximum residue limits in foodstuffs of animal origin. 2009.

[6] Falowo AB, Akimoladun OF. Veterinary drug residues in meat and meat products: occurence, detection and implications. Veterinary Pharmaceuticals: IntechOpen. 2019.

[7] Olivares IRB, Souza GB, Nogueira ARA, Toledo GTK, Marcki DC (2018). Trends in developments of certified reference materials for chemical analysis - focus on food, water, soil, and sediment matrices. TrAC, Trends Anal Chem.

[8] CFIA Meat Hygiene Directives, Chapter 5, Section 5.2.1.1. 2018.

[9] ISO 17034: General Requirements for the Competence of Reference Material Producers, International Organization for Standardization, Brussels, Belgium. 2016.

[10] Violante FGM, Rosas CdO, Guimarães EdF, Vital HdC, Zúniga NOC, Aquino Neto FRd (2018). Feasibility study for the development of a certified reference material of nitrofuran metabolites in chicken breast muscle from incurred samples. Measurement..

[11] Zeleny R, Schimmel H, Emtoberg H, Emons H. Certification Report: The Certification of the Mass Fraction of Chloramphenicol in Pork Meat, Certified Reference Material ERM-BB130. Geel, Belgium: European Commission, Joint Research Research Center, Institute for Reference Materials and Measurements. 2008.

[12] Zeleny R, Schimmel H, Ulberth F, Emons H. Certification Report: Certification of Mass Fractions of Nitroimidazoles in Pork Meat, Certified Reference Materilas ERM-BB124. Geel, Belgium: European Commission, Joint Research Center, Institute for Reference Materials and Measurements. 2008.

[13] Li F, Zhou J, Wang M, Zhang L, Yang M, Deng L (2023). Production of a matrix certified reference material for measurement and risk monitoring of clenbuterol in mutton. Anal Bioanal Chem.

[14] Windust A, McRae G, Meija J, Mester Z, Melanson JE, Croft M (2019). High polarity analytes in food - enrofloxacin and sulfadiazine in bovine tissue (CCQM-K141). Metrologia..

[15] Canadian Council on Animal Care, Guide to the Care and Use of Experimental Animals, Volume 1, 2nd Edition, (1993, Revised in 2020). Available from https://ccac.ca/Documents/Standards/Guidelines/Experimental_Animals_Vol1.pdf. Accessed on 12 Aug 2020

[16] Wang Z, Hu S, Bao H, Xing K, Liu J, Xia J (2021). Immunochromatographic assay based on time-resolved fluorescent nanobeads for the rapid detection of sulfamethazine in egg, honey, and pork. J Sci Food Agric.

[17] Yu L, Liu M, Wang T, Wang X (2019). Development and application of a lateral flow colloidal gold immunoassay strip for the rapid quantification of ciprofloxacin in animal muscle. Anal Methods.

[18] Aboubakr MH (2013). Evaluation of bioequivalence of two enrofloxacin formulations after intramuscular administration in goats. Koream J Vet Res.

[19] Castillo-Aguirre A, Cañas A, Honda L, Richter P (2021). Determination of veterinary antibiotics in cow milk using rotating-disk sorptive extraction and liquid chromatography. Microchem J.

[20] Monteiro SH, Lehotay SJ, Sapozhnikova Y, Ninga E, Lightfield AR (2021). High-throughput mega-method for the analysis of pesticides, veterinary drugs, and environmental contaminants by ultra-high-performance liquid chromatography-tandem mass spectrometry and robotic mini-solid-phase extraction cleanup + low-pressure gas chromatography-tandem mass spectrometry, part 1: beef. J Agric Food Chem.

[21] Na TW, Seo HJ, Jang SN, Kim H, Yun H, Kim H (2022). Multi-residue analytical method for detecting pesticides, veterinary drugs, and mycotoxins in feed using liquid- and gas chromatography coupled with mass spectrometry. J Chromatogr A.

[22] Lehotay SJ, Lightfield AR (2021). Comparison of four different multiclass, multiresidue sample preparation methods in the analysis of veterinary drugs in fish and other food matrices. Anal Bioanal Chem.

[23] Medellín-Martínez MF, Luna-Zavala I, Martínez-Delgado M, Pérez-Urizar JT, Ramírez-Telles JA, Patiño-Rodríguez O (2018). Sensitive assay of clenbuterol residues in beef by ultra-high performance liquid chromatography coupled with mass spectrometry (UPLC-MS/MS) and solid-phase extraction. Food Anal Methods.

[24] Zhao L, Lucas D, Long D, Richter B, Stevens J (2018). Multi-class multi-residue analysis of veterinary drugs in meat using enhanced matrix removal lipid cleanup and liquid chromatography-tandem mass spectrometry. J Chromatogr A.

[25] Magalhaes D, Freitas A, Sofia Vila Pouca A, Barbosa J, Ramos F (2020). The use of ultra-high-pressure-liquid-chromatography tandem time-of-flight mass spectrometry as a confirmatory method in drug residue analysis: application to the determination of antibiotics in piglet liver. J Chromatogr B Analyt Technol Biomed Life Sci..

[26] Mehl A, Hudel L, Bucker M, Morlock GE (2022). Validated screening method for 81 multiclass veterinary drug residues in food via online-coupling high-throughput planar solid-phase extraction to high-performance liquid chromatography-orbitrap tandem mass spectrometry. J Agric Food Chem.

[27] Wang H, Tian H, Ai LF, Liang SX (2023). Screening and quantification of 146 veterinary drug residues in beef and chicken using QuEChERS combined with high performance liquid chromatography-quadrupole orbitrap mass spectrometry. Food Chem.

[28] McCarron P, Giddings SD, Reeves KL, Hess P, Quilliam MA (2015). A mussel (Mytilus edulis) tissue certified reference material for the marine biotoxins azaspiracids. Anal Bioanal Chem.

[29] McCarron P, Wright E, Emteborg H, Quilliam MA (2017). A mussel tissue certified reference material for multiple phycotoxins. Part 4: certification. Anal Bioanal Chem..

[30] Nadeau K, Mester Z, Yang L (2018). The direct and accurate determination of major elements Ca, K, Mg and Na in water by HR-ICPMS. Sci Rep.

[31] Pagliano E, Meija J, Campanella B, Onor M, Iammarino M, D’Amore T (2019). Certification of nitrate in spinach powder reference material SPIN-1 by high-precision isotope dilution GC-MS. Anal Bioanal Chem.

[32] Bates J, Bahadoor A, Cui Y, Meija J, Windust A, Melanson JE. Certification of ochratoxin A reference materials: calibration solutions OTAN-1 and OTAL-1 and a mycotoxin-contaminated rye flour MYCO-1. J AOAC Int. 2019, 102(6):1756-1766. 10.5740/jaoacint.19-0086. Epub 26 Aug 2019 10.5740/jaoacint.19-0086 31451132

[33] Meija J, Mester Z (2008). Paradigms in isotope dilution mass spectrometry for elemental speciation analysis. Anal Chim Acta.

[34] Vogl J (2012). Measurement uncertainty in single, double and triple isotope dilution mass spectrometry. Rapid Commun Mass Spectrom.

[35] Vogl J, Pritzkow W. Isotope dilution mass spectrometry - a primary method of measurement and its role for RM certification. MAPAN J Metrol Soc India. 2010;25(3):135–64.

[36] Pagliano E, Meija J (2016). Reducing the matrix effects in chemical analysis: fusion of isotope dilution and standard addition methods. Metrologia.

[37] Health of Animals Regulation, PartXIV - Food for Ruminants, Livestock and Poultry, Rendering Plants, Fertilizer and Fertilizer Supplements. 2012. https://lawslois.justice.gc.ca/eng/regulations/c.r.c.,_c._296/page-13.html#h-548290. Accessed on 12 Aug 2020

[38] Melanson JE, Thibeault MP, Stocks BB, Leek DM, McRae G, Meija J (2018). Purity assignment for peptide certified reference materials by combining qNMR and LC-MS/MS amino acid analysis results: application to angiotensin II. Anal Bioanal Chem.

[39] Pagliano E, Mester Z, Meija J (2013). Reduction of measurement uncertainty by experimental design in high-order (double, triple, and quadruple) isotope dilution mass spectrometry: application to GC-MS measurement of bromide. Anal Bioanal Chem.

[40] Meija J, McRae G, Pagliano E (2020). Application of regression methods to solve general isotope dilution measurement equations. Metrologia.

[41] Schoenberger T (2012). Determination of standard sample purity using the high-precision 1H-NMR process. Anal Bioanal Chem.

[42] Saito T, Ihara T, Koike M, Kinugasa S, Fujimine Y, Nose K (2008). A new traceability scheme for the development of international system-traceable persistent organic pollutant reference materials by quantitative nuclear magnetic resonance. Accred Qual Assur.

[43] Nelson MA, Bedner M, Lang BE, Toman B, Lippa KA (2015). Metrological approaches to organic chemical purity: primary reference materials for vitamin D metabolites. Anal Bioanal Chem.

[44] Geis-Asteggiante L, Lehotay SJ, Lightfield AR, Dutko T, Ng C, Bluhm L (2012). Ruggedness testing and validation of a practical analytical method for >100 veterinary drug residues in bovine muscle by ultrahigh performance liquid chromatography-tandem mass spectrometry. J Chromatogr A.

[45] Schneider MJ, Lehotay SJ, Lightfield AR (2012). Evaluation of a multi-class, multi-residue liquid chromatography-tandem mass spectrometry method for analysis of 120 veterinary drugs in bovine kidney. Drug Test Anal.

[46] Martos PA, Jayasundara F, Dolbeer J, Jin W, Spilsbury L, Mitchell M (2010). Multiclass, multiresidue drug analysis, including aminoglycosides, in animal tissue using liquid chromatography coupled to tandem mass spectrometry. J Agric Food Chem.

[47] Fuh M-RS, Chan S-A (2001). Quantitative determination of sulfonamide in meat by liquid chromatography-electrospray-mass spectrometry. Talanta..

[48] Jedziniak P, Szprengier-Juszkiewicz T, Olejnik M (2009). Multi-residue screening method for the determination of non-steroidal anti-inflammatory drug residues in cow’s milk with HPLC-UV and its application to meloxicam residue depletion study. Bell Vet Inst Pulawy.

[49] ISO Guide 35: Reference Materials - Guidance for Characterization and Assessment of Homogeneity and Stability, International Organization for Standardization, Brussels. Belgium, 2017.

[50] DerSimonian R, Laird N (1986). Meta-analysis in clinical trials. Control Clin Trials.

[51] Viechtbauer W. metafor: meta-analysis package for R 2020 [Available from: https://CRAN.R-project.org/package=metafor. Accessed 12 Aug 2020

[52] Horwitz W (1982). Evaluation of analytical methods used for regulation of foods and drugs. Anal Chem.

[53] Sieber JR, Epstein MS, Possolo AM. A retuned Horwitz procedure for upgrading certificates of older standard reference materials. United States Department of Commerce, National Institute of Standards and Technology Special publication. 2019;260–198. 10.6028/NIST.SP.260-198. Accessed on 19 May 2020

[54] JCGM 100:2008. Evaluation of Measurement Data - Guide to the Expression of Uncertainty in Measurement. 2008.

