# Supplementary material for: Production and certification of BOTS-1: bovine muscle–certified reference material for incurred veterinary drug residues
Source: Anal Bioanal Chem. 2023 Jun 16;416(3):759–71. doi: 10.1007/s00216-023-04794-5 (PMC10766801; doi:10.1007/s00216-023-04794-5)
Supplement: Supplementary file 1 — Supplementary file1 (DOCX 462 KB) [file 216_2023_4794_MOESM1_ESM.docx]

**Production and certification of BOTS-1: Bovine muscle certified reference material for incurred veterinary drug residues**

Garnet McRae,^1,^* Donald M. Leek,^1^ Juris Meija,^1^ Bryn Shurmer,^2^ Steven J. Lehotay,^3^

Joachim Polzer,^4^ Jeremy E. Melanson,^1^ and Zoltan Mester ^1^

^1^ National Research Council Canada, Metrology, 1200 Montreal Road, Ottawa, ON K1A 0R6 Canada

^2^ Canadian Food inspection Agency, 116 Veterinary Road, Saskatoon, Saskatchewan

^3^ US Department of Agriculture, Agricultural Research Service, Eastern Regional Research Center, 600 East Mermaid Lane, Wyndmoor, PA 19038, USA

^4^ German Federal Office of Consumer Protection and Food Safety (BVL), Mauerstrasse 39-42, 10117, Berlin, Germany

*Corresponding author’s email: [garnet.mcrae@nrc-cnrc.gc.ca](mailto:garnet.mcrae@nrc-cnrc.gc.ca)

**Table of Contents**

**List of Tables**

**Table S-1 Veterinary drugs dosed in the production of BOTS-1**

**Table S-2 Veterinary drug residue mass fraction results for all laboratories. (Results provided by laboratories including NRC, CFIA, USDA, BVL and CCQM K141/P178 participants)**

**List of Figures**

**Figure S-1 Extraction time - Peak area: MR-1**

**Figure S-2 Extraction time - Peak area ratio: MR-1**

**Figure S-3 Serial extraction: MR-1**

**Figure S-4 Extraction time - Peak area: MR-2**

**Figure S-5 Extraction time - Peak area ratio: MR-2**

**Figure S-6 Serial extraction: MR-2**

**Figure S-7 Correlation between relative difference in ID^2^MS and SA-ID^2^-MS results and matrix effect**

**Figure S-8 Chlorpromazine - homogeneity**

**Figure S-9 Ciprofloxacin - homogeneity**

**Figure S-10 Clenbuterol - homogeneity**

**Figure S-11 Dexamethasone - homogeneity**

**Figure S-12 Enrofloxacin - homogeneity**

**Figure S-13 Meloxicam - homogeneity**

**Figure S-14 Ractopamine - homogeneity**

**Figure S-15 Sulfadiazine - homogeneity**

**Figure S-16 Short-term stability: MR-1**

**Figure S-17 Short-term stability: MR-2**

**Figure S-18 Freeze-thaw stability: MR-1**

**Figure S-19 Freeze-thaw stability: MR-2**

**Figure S-20 Long-term stability: MR-1**

**Figure S-21 Long-term stability: MR-2**

**Table S-1** Veterinary drugs dosed in the production of BOTS-1 (MRL = maximum residue limit; MMPR = minimum method performance requirements)

| **Drug** | **Usage** | **Health**  **Canada**  **Status** | **MRL/MMPR**  **(ng/g)** | **Dose**  **type** | **Dose**  **(mg/kg)** | **Dosing**  **prior to**  **euthanasia** |
| --- | --- | --- | --- | --- | --- | --- |
| chloramphenicol | antibiotic | banned | MMPR: 0.3 | injection | 2 | 2 days |
| Enrofloxacin^1^ | antibiotic | allowed | MRL: 20 | injection | 5 | 2 days |
| nitrofurantoin | antibiotic | banned | MMPR: 5 | oral | 2 | 4 days |
| sulfadiazine | antibiotic | allowed | MRL: 200 | injection | 20 | 1 day |
| Tulathromycin^2^ | antibiotic | allowed | MRL: 1000 | injection | 2.5 | 2 days |
| trimethoprim | antibiotic | allowed | MRL: 50 | injection | 0.5 | 2 days |
| ractopamine | growth promoter | allowed | MRL: 20 | feed | 30 | 7 days |
| clenbuterol | growth promoter | banned | MMPR: 0.5 | injection | 0.002 | 4 hours |
| dexamethasone | anti-inflammatory | allowed | MRL: 0.5 | injection | 0.05 | 1 day |
| trenbolone | growth promoter | allowed | MRL: 2 | implant | 4 implants | 7 days |
| meloxicam | pain reliever | allowed | MRL: 20 | injection | 0.3 | 4 days |
| Phenylbutazone^3^ | pain reliever | no indications | MMPR: 5 | injection | 4.4 | 7 days |
| chlorpromazine | tranquilizer | no indications | MMPR: 10 | injection | 0.5 | 4 hours |

^1^ Ciprofloxacin metabolite produced in-vivo

^2^ CP-60,300 metabolite produced in-vivo

^3^ Oxyhenbutazone metabolite produced in-vivo

**Table S-2**  Veterinary drug residue mass fraction results for all laboratories. (Results provided by laboratories including NRC, CFIA, USDA, BVL and CCQM K141/P178 participants)

| Lab | CProm | | Cipro | | Clen | | Dexa | | Enro | | Meloxi | | Racto | | Sulfa | |
| --- | --- | --- | --- | --- | --- | --- | --- | --- | --- | --- | --- | --- | --- | --- | --- | --- |
|  | w_A_ | *u* | w_A_ | *u* | w_A_ | *u* | w_A_ | *u* | w_A_ | *u* | w_A_ | *u* | w_A_ | *u* | w_A_ | *u* |
|  | (ng/g) | | (ng/g) | | (ng/g) | | (ng/g) | | (ng/g) | | (ng/g) | | (ng/g) | | (ng/g) | |
| NRC-1 | 537 | 20 | 43.9 | 2.7 | 3.74 | 0.28 | 9.09 | 0.66 | 52.1 | 2.2 | 3.05 | 0.24 | 11.9 | 0.8 | 2376 | 65 |
| NRC-2 | 450 | 19 | 43.5 | 2.9 | 3.59 | 0.30 | 9.09 | 0.72 | - | - | 3.07 | 0.24 | 12.7 | 0.8 | - | - |
| Ex-1 | 378 | 56 | 55.4 | 13.4 | - | - | 9.26 | 2.90 | 53.5 | 13.6 | 2.87 | 0.60 | 12.8 | 1.5 | 2217 | 388 |
| Ex-2 | 396 | 86 | 44.0 | 6.0 | 4.40 | 1.80 | 10.0 | 0.5 | 56.0 | 7.0 | 3.10 | 0.60 | 13.0 | 2.4 | - | - |
| Ex-3 | - | - | 52.4 | 5.3 | 2.45 | 0.25 | 9.34 | 1.0 | 59.3 | 6.9 | - | - | - | - | 2292 | 199 |
| CCQM-1 | - | - | - | - | - | - | - | - | 51.8 | 3.3 | - | - | - | - | 2243 | 128 |
| CCQM-2 | - | - | - | - | - | - | - | - | 53.0 | 0.8 | - | - | - | - | 2207 | 69 |
| CCQM-3 | - | - | - | - | - | - | - | - | 53.4 | 1.7 | - | - | - | - | 2235 | 69 |
| CCQM-4 | - | - | - | - | - | - | - | - | 53.6 | 1.8 | - | - | - | - | 2364 | 66 |
| CCQM-5 | - | - | - | - | - | - | - | - | 54.7 | 1.5 | - | - | - | - | 2361 | 76 |
| CCQM-6 | - | - | - | - | - | - | - | - | 58.8 | 2.4 | - | - | - | - | 2398 | 96 |
| CCQM-7 | - | - | - | - | - | - | - | - | 59.0 | 3.3 | - | - | - | - | 2235 | 127 |
| CCQM-8 | - | - | - | - | - | - | - | - | 59.0 | 2.7 | - | - | - | - | 2269 | 99 |
| CCQM-9 | - | - | - | - | - | - | - | - | 61.7 | 1.9 | - | - | - | - | 2127 | 69 |
| CCQM-10 | - | - | - | - | - | - | - | - | 62.2 | 2.2 | - | - | - | - | 2313 | 58 |
| CCQM-11 | - | - | - | - | - | - | - | - | 64.8 | 2.7 | - | - | - | - | 2337 | 78 |
| CCQM-12 | - | - | - | - | - | - | - | - | 65.5 | 3.8 | - | - | - | - | 2521 | 118 |
| CCQM-13 | - | - | - | - | - | - | - | - | 57.7 | 3.0 | - | - | - | - | - | - |
| Mean * | 490 |  | 44 |  | 3.3 |  | 9.5 |  | 57 |  | 3.0 |  | 12.4 |  | 2290 |  |
| *u*c ** | 50 |  | 2.2 |  | 0.7 |  | 0.4 |  | 2.4 |  | 0.2 |  | 0.6 |  | 60 |  |
| *U*c *** | 100 |  | 4.4 |  | 1.4 |  | 0.8 |  | 4.8 |  | 0.4 |  | 1.2 |  | 120 |  |

NRC-1: ID^2^-MS

NRC-2: SA-ID^2^-MS

Ex: External laboratories

CCQM: K141/P178 participating laboratories

* Bayesian Gaussian Random Effects Model (REM)

** Combined uncertainty including homogeneity, stability and characterization (measurement, primary standards and dark uncertainty)

*** Expanded uncertainty using K=2

**Figure S-1** Extraction time evaluation: Analyte peak area, relative to peak area at 60 minutes, versus extraction time

MR-1: Cipro, Clen, Enro, Racto, Sulfa

**Figure S-2** Extraction time evaluation: Analyte/Internal standard peak area ratio, relative to peak area ratio at 60 minutes, versus extraction time

MR-1: Cipro, Clen, Enro, Racto, Sulfa

**Figure S-3** Serial extraction evaluation: Cumulative analyte peak area versus number of serial extractions

MR-1: Cipro, Clen, Enro, Racto, Sulfa

**Figure S-4** Extraction time evaluation: Analyte peak area, relative to peak area at 60 minutes, versus extraction time

MR-2: Cprom, Dexa, Meloxi

**Figure S-5** Extraction time evaluation: Analyte/Internal standard peak area ratio, relative to peak area ratio at 60 minutes, versus extraction time

MR-2: Cprom, Dexa, Meloxi

**Figure S-6** Serial extraction evaluation: Cumulative analyte peak area versus number of serial extractions

MR-2: Cprom, Dexa, Meloxi

**Figure S-7** Correlation between relative difference in ID^2^MS and SA-ID^2^-MS mass fraction results and matrix effect for method multi-residue-1 (MR-1) and multi-residue-2 (MR-2) for methods multi-residue-1 (MR-1) and multi-residue-2 (MR-2)

**Figure S-8** Chlorpromazine mass fraction homogeneity results with error bars representing the standard uncertainties of duplicate sample results

**Figure S-9** Ciprofloxacin mass fraction homogeneity results with error bars representing the standard uncertainties of duplicate sample results

**Figure S-10** Clenbuterol mass fraction homogeneity results with error bars representing the standard uncertainties of duplicate sample results

**Figure S-11** Dexamethasone mass fraction homogeneity results with error bars representing the standard uncertainties of duplicate sample results

**Figure S-12** Enrofloxacin mass fraction homogeneity results with error bars representing the standard uncertainties of duplicate sample results

**Figure S-13** Meloxicam mass fraction homogeneity results with error bars representing the standard uncertainties of duplicate sample results

**Figure S-14** Ractopamine mass fraction homogeneity results with error bars representing the standard uncertainties of duplicate sample results

**Figure S-15** Sulfadiazine mass fraction homogeneity results with error bars representing the standard uncertainties of duplicate sample results

**Figure S-16** Short-term stability (2 weeks): Mass fraction relative to -80 °C control samples versus temperature

MR-1: Cipro, Clen, Enro, Racto, Sulfa

**Figure S-17** Short-term stability (2 weeks): Mass fraction relative to -80 °C control samples versus temperature

MR-2: Cprom, Dexa, Meloxi

**Figure S-18** Freeze-thaw stability: Mass fraction relative to one freeze-thaw cycle versus number of freeze-thaw cycles

MR-1: Cipro, Clen, Enro, Racto, Sulfa

**Figure S-19** Freeze-thaw stability: Mass fraction relative to one freeze-thaw cycle versus number of freeze-thaw cycles

MR-2: Cprom, Dexa, Meloxi

**Figure S-20** Long-term stability: Mass fraction relative to time zero versus storage time at -80 °C

MR-1: Cipro, Clen, Enro, Racto, Sulfa

**Figure S-21** Long-term stability: Mass fraction relative to time zero versus storage time at -80 °C

MR-2: Cprom, Dexa, Meloxi
